# Assessment of Physical Well-Being and Leisure Time of Polish Students during the COVID-19 Outbreak

**DOI:** 10.3390/ijerph19148358

**Published:** 2022-07-08

**Authors:** Sylwia Jaskulska, Barbara Jankowiak, Mateusz Marciniak, Michal Klichowski

**Affiliations:** Faculty of Educational Studies, Adam Mickiewicz University, 60-568 Poznan, Poland; barbara.jankowiak@amu.edu.pl (B.J.); mateusz.marciniak@amu.edu.pl (M.M.); klichowski.michal@gmail.com (M.K.)

**Keywords:** distance education, free time, physical comfort, SARC-CoV-02, school experiences

## Abstract

This project aims to recognize the school experiences of Polish students during the COVID-19 outbreak; we focused on the area of assessment of physical well-being and leisure time. Nearly two thousand primary or secondary school students aged 9 to 20 participated in the survey. Running descriptive statistics, frequency analysis, and significant difference tests, we found that 45% of students thought their physical well-being during the COVID-19 outbreak worsened compared to pre-pandemic times. Boys declared they felt better in their physical well-being than girls (*p* < 0.001). Most students noticed changes in the quality of their leisure time activities; boys were happier than girls in their free time during the outbreak (*p* < 0.001). Learners’ ages also differentiated the assessment of their physical well-being (*p* < 0.001) and leisure time (*p* < 0.001). The youngest students more often assessed their experiences in the researched fields as good or increased, while the older groups more often noticed a decrease. Multinomial logistic regression proved that the differences in the assessments of physical well-being and leisure time could be explained to the greatest extent by age and gender, whereas the place of residence and school location were insignificant. All in all, our study confirms the dominance of the adverse effects of the COVID-19 pandemic on physical well-being and changes in leisure time. As such, it is advisable (during and after outbreaks) to support children and adolescents by targeting individual and institution levels. We recommend developing techniques to reduce stress and information overload, increasing creative ways of spending leisure time, supporting families in navigating children’s free time, and expanding social support networks.

## 1. Introduction

Restrictions introduced in many countries to reduce the effects of the spread of SARC-CoV-02, which causes the COVID-19 disease, have changed the organization of everyday life and the structure of fundamental areas of activity (e.g., work, education, and free time). The strategies were mainly related to hygiene behavior (wearing face masks, disinfecting hands) and minimizing viral emissions by limiting social contact. Temporary restrictions on the possibilities of movement, gatherings, and meetings and reduced access to public space, including traditional entertainment facilities (e.g., sports or cultural), have changed the way children and adolescents, as well as adults, spend their free time, including their physical activity. The modifications in habits forced on them by the pandemic indicate an increase in time spent at home, a focus on entertainment mediated by social media (games, streaming, TV), and the growing importance of individually undertaken outdoor activities [1,2,3].

These changes affected all social groups but not to the same extent—there are socioeconomic and demographic differences in this respect. For example, studies have demonstrated intergenerational differences in responding to the pandemic in terms of leisure activities. Among adults, the number of activities related to work, learning, cooking, watching movies and series (TV, stream), social media video-chatting, reading, physical activities, and household chores increased the most during the COVID-19 pandemic [4]. For Spanish adults, a particular increase was recorded in areas such as reading for pleasure (54.5%), talking and other activities with household members (51.5%), and playing board games (37.0%) but also in doing nothing (40.1%). As far as physical activity is concerned, it was undertaken by 4 out of 10 respondents more often than before the pandemic and less often by 3 in 10 respondents. From the perspective of gender, men indicated less physical exercise (43.2%), and women more (44.6%). In an Italian study [2], a comparison between young people (between 12 and 24 years of age) and older people (between 55 and 64 years) showed that among the first group, clearly more people indicated an increase in the frequency of watching TV programs (an increase among 72.5% of respondents compared to 46.0% in the older group) and indoor physical activity (58.4% compared to 29.2%), as well as in idle time (54.0% compared to 34.4%), listening to music (53.1% compared to 34.0%), and artistic activities or hobbies (48.0% compared to 31.5%). Young people also indicated more significant changes in the satisfaction of basic needs during the lockdown period: They slept more (51.4% versus 26.7% of respondents aged 55–64) and ate more (48.5% versus 35.2%) and increased the time spent on beauty treatments (27.7% versus 9.1%).

Before the pandemic, children and adolescents under 18 most often undertook activities related to peer and social interactions in their free time in dedicated entertainment venues [2,3]. Among the factors related to barriers to entertainment activity in research on a representative group of people over 16 in Great Britain, the following were identified: mental difficulties (e.g., depression, anxiety), household chores and less time for entertainment, lack of resources (e.g., lack of funds), limitations in transport, and limitations in the sphere of social interactions (e.g., closed places of entertainment and limitations in school life) [5]. The results of a Dutch study showed that before the pandemic, children and adolescents most often undertook activities in their free time, such as going to the cinema and clubs and participating in sports events and shopping [3]. The ways of spending time were related to social/cultural events that were stopped during the pandemic, forcing the entertainment industry to adapt [3]. Traditional forms of spending free time in places of entertainment lost their importance as a result of the pandemic as they were based on mobility and social mobility [2,6,7]. On the other hand, forms of entertainment mediated by the media have increased, which has led to the development of new strategies and forms of spending free time among young people [1,8]. This is not surprising because the media is an essential source of information and entertainment for young people [6]. The impact of media, including social media, on leisure activities and individual well-being during the COVID-19 pandemic has been overwhelming and ambiguous. Media can increase stress and cause increased anxiety due to exposure to an excess of negative information on social media or news websites [1,9,10]. Such exposure causes negative consequences at both the individual level (e.g., reduced mental well-being and information overload) and the social level (e.g., overload of the health care system as a result of the “panic” of media recipients) [1,11,12]. Moreover, research on young people aged 18–23 [8] has shown that information overload (difficulties in processing and dealing with information) due to social media use is correlated with lower levels of mental well-being and higher levels of COVID-19 anxiety, discouragement, and media fatigue.

Changes in the habits of children and adolescents observed during the pandemic may contribute to transformations in terms of lifestyle [1,2,3] and modifications in the level of well-being [8,10,13], social relations [3,14], identity development, orientation, and motivation [3,15]. Interestingly, some research on the transformation of leisure activities and well-being in the COVID-19 period has signaled potential positive changes for the development of children and adolescents. The amount of unstructured leisure time (replacing structured time) increased significantly, which may be a pro-development factor, requiring independence and initiative [2]. During the pandemic, an increase in activities aimed at independent learning [2] and a more generalized increase in creative activity [5] were observed. People who spent relatively more free time on creative endeavors (broadly understood as arts and culture), sports, and language learning showed better well-being during the COVID-19 pandemic. Motivations focused on seeking creative expression, cognitive stimulation, good physical condition, and social relationships were associated with better well-being [5].

Short and long-term consequences of the COVID-19 pandemic on the development of children and adolescents are expected. Therefore, at the initial pandemic stage, in their “#HealthyAtHome—Mental health” campaign [10], the World Health Organization made recommendations regarding free time and its relationship to well-being. Physical well-being achieved by daily routine activities (including regular sleep, hygiene, healthy eating, and routine exercise) has been identified as an important factor in mental health [10]. Among the strategies recommended by WHO as potential factors protecting against the negative effects of the pandemic, there are many related to the way of spending free time. They concern, for example, social activities for the benefit of others, keeping a balance between work and family time, and finding time for favorite activities, but also avoiding stimulants, excessive watching of TV, or playing computer games [10].

How the COVID-19 outbreak influenced the assessment of Polish students’ physical well-being and leisure time is an open question. It cannot be assumed that the effects of the outbreak in Poland were identical to those observed in other countries. In Poland, compared to other European countries, there is low vaccine acceptance and low trust in health professionals, doctors, nurses, pharmacists, and national health authorities. There was also lower resistance to following the anti-COVID recommendations [16]. Moreover, the organization of school education in Poland during the COVID-19 outbreak was specific. In short, students from the first three years of primary school (aged 6 to 9/10 years) had a relatively short duration of distance learning. Older students in primary school (9/10 to 15 years) and secondary school learners (aged 15 to 20) studied mainly online. Nevertheless, most of the time, from April 2020, activities for children and teenagers under 18 were minimal, e.g., only the presence of a parent, legal guardian, or an adult could justify their presence outside the home [17]. Therefore, while conducting an extensive survey aimed at the school experiences of Polish learners during the COVID-19 pandemic (VULCAN Project), we also asked them about these issues. (This was when students aged 9/10 to 20 were slowly transitioning from total distance learning, lasting 7 months, to a hybrid version of learning but before they fully return to offline learning [18].) In this paper, we present the outcomes of that part of the research (for more results, see [14,19,20,21]). Our findings confirm the existence of demographic differences in the effects of the outbreak on physical well-being and the assessment of the quality of free time. We discuss which groups should receive exceptional support after the pandemic (or after each wave) to resolve the deepening differences. Thus, our results fill the knowledge gap in the field of Polish high-risk groups related to outbreak effects.

## 2. Materials and Methods

### 2.1. Participants

A large sample of Polish students (*N* = 1955) aged between 9 and 20 participated in the study (14.1% 9–12 years, 30.2% 13–15 years, 33.5% 16–17 years, 13.5% 18–20 years, 8.8% no declaration). We aimed to study more than 1000 students, as previous studies (e.g., [22]) have demonstrated that such a sample size allows for some generalizations regarding the functioning of Polish students. However, *N* = 1000 is a much larger sample than required. In the school year 2020/2021, 4.931.461 learners studied at all levels of schools in Poland. Thus, with a fraction size of 0.5 (50%), a maximum error of 5%, and a confidence level of 95% (*α* = 0.95), the minimum sample size would be 384 people (https://www.naukowiec.org/dobor.html, accessed on 16 June 2022).

All participants were volunteers and users of one of the most extensive electronic school diaries used in Poland (VULCAN, www.vulcan.edu.pl, accessed on 12 May 2022) and attended primary (second level) or secondary schools. The study sample was balanced in terms of essential characteristics, such as gender (43.3% girls, 46.2% boys, 10.5% no declaration), place of residence (the respondents came from all Polish voivodeships; 40.4% lived in the countryside, 34.4% in small towns, and 24.8% in large cities), and school location (14.6% studied in schools located in the countryside, 51.9% in small towns, and 33.5% in large cities).

### 2.2. Procedure

The survey was conducted in accordance with the principles of the Helsinki Declaration. The participants were informed about the purpose of the research, their rights, and the confidentiality of the collected data. Informed consent was obtained from all of them or their parents or legal guardians.

The questionnaire was implemented in the *WebAnkieta* environment (www.webankieta.pl, accessed on 1 May 2021) and was active at the end of May and the beginning of June 2021. Students, their parents, or legal guardians received a message via the VULCAN diary describing the study and asking them (or their child) to complete an online questionnaire. Volunteers completed it at home in their own time. The questionnaire consisted of 26 questions and was divided into three parts: (1) My school life now, during the pandemic, (2) my school life before and during the pandemic, and (3) my school after the pandemic. In this work, we analyze answers to the closed questions regarding the assessment of physical well-being (“How do you rate your physical well-being before the pandemic and during distance education?”) and the way of spending leisure time (“Free time allows us to rest, develop passions, take care of our bodies and minds. How would you rate your leisure activities before the pandemic and during distance education?”) during the pandemic. Analyses of answers to other questions (regarding other aspects of the pandemic) are included in our other reports [14,19,20,21] and will appear in subsequent ones.

The questionnaire also included a socio-demographic section with questions about gender, age, place of residence, and school location. We utilized questions with a closed list of answers/options.

Before the start of the study, the questionnaire was sent to two children aged 10 years. Each child was to read all the questions. After reading them, we conducted online interviews with them, in which we checked whether the questions could be understood. Based on these interviews, we made minor corrections to the questionnaire so that it was readable for all respondents.

### 2.3. Data Analysis

The two dependent variables were measured with a nominal scale; the independent variables (predictors) were measured with a nominal or ordinal scale. Thus, we analyzed them mainly by percentage—we used descriptive statistics, frequency analysis, and the significant difference chi-square test (*ch*^2^). For analyzing the relation between dependent variables and multiple independent variables, we used a multinomial logistic regression model (with age, gender, place of residence, and school location), followed by subgroup analysis (by age and gender) with chi-square tests. The adopted level of significance (*p*) was *α* = 0.05 (1−*α* = 0.95). For all statistical analyses, we used IBM^®^ SPSS Statistics^®^ for Windows Version 27.0 (IBM Corp., Armonk, NY, USA).

## 3. Results

**Figure 1:**
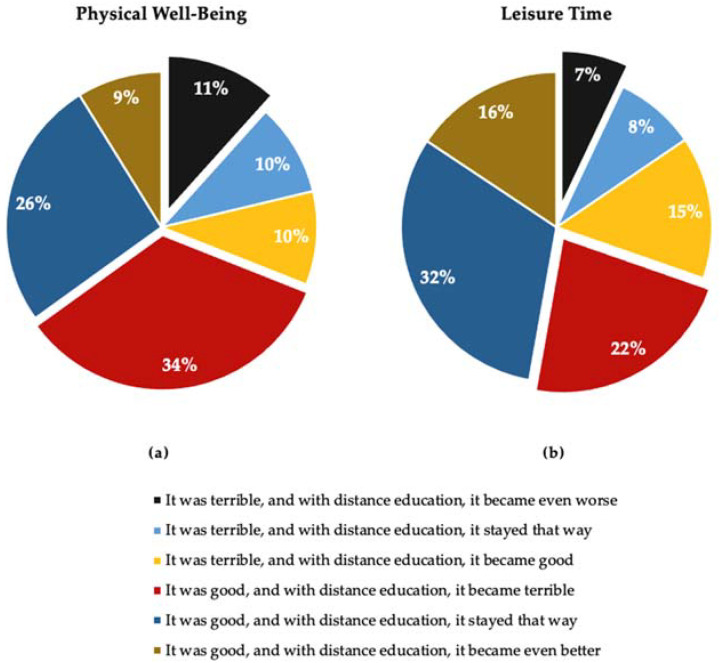
Descriptive results of the study. (**a**) Assessment of physical well-being during the COVID-19 outbreak in the experiences of Polish students: almost half of the participants declared deterioration in this area (black and maroon parts), and one-fourth did not notice any changes (navy blue). (**b**) Assessment of leisure time spending during the COVID-19 outbreak in the experiences of Polish students: one-third of the participants declared a deterioration here (black and maroon part); at the same time, one-third did not notice any change (navy blue). *N* = 1955.

As shown in Figure 1a, 45% of participants declared that their physical well-being had deteriorated when comparing the distance education period to the earlier period (they chose the answers: “It was terrible, and with distance education, it became even worse” or “It was good, and with distance education, it became terrible”). The answer “It was good, and with distance education, it stayed that way” was chosen by more than 26% of students and “It was terrible, and with distance education, it stayed that way” by over 9%. Thus, over 35% of students did not experience any change in well-being during the pandemic. Interestingly, about 19% of the students thought that their physical well-being improved (they chose “It was terrible, and with distance education, it became good” or “It was good, and with distance education, it became even better”).

Regarding leisure time (see Figure 1b), nearly 30% of participants noticed negative changes in how they spent it during distance education (they chose answers such as “It was terrible, and with distance education, it became even worse” or “It was good, and with distance education, it became terrible”). A similar number of respondents (about 30%) believed that their way of spending free time had improved (they noted: “It was terrible, and with distance education, it became good” or “It was good, and with distance education, it became even better”). However, almost 40% said that their leisure time had not changed for the better or worse during distance education (they chose: “It was terrible, and with distance education, it stayed that way” or “It was good, and with distance education, it stayed that way”).

### 3.1. Physical Well-Being and Leisure Time—Differences in Gender, Age, Place of Residence, and School Location

Gender significantly differentiated the experiences of the surveyed students during distance education (see Table 1). Regarding the self-assessment of their physical well-being (top panel in Table 1), boys declared that they felt better than girls (*ch*^2^ = 18.231, *df* = 5, *p* < 0.001). The answer that the well-being “was good, and with distance education, it stayed that way” was chosen by 29.7% of boys and only 24.6% of girls; “it was good, and with distance education, it became even better” was chosen by 10.0% of boys and 7.6% of girls, while “it was terrible, and with distance education, it stayed that way” was chosen by 7.3% of boys and 11.0% of girls. Additionally, the issue of spending leisure time was assessed more positively by boys than girls (*ch*^2^ = 18.653, *df* = 5, *p* < 0.001; bottom panel in Table 1). The answer that free time “was good, and with distance education, it became terrible” was chosen by 20.0% of boys and 26.6% of girls, while “it was good, and with distance education, it became even better” was chosen by 18.5% of boys and only 13.1% of girls.

Age also differentiated the assessment of one’s own physical well-being (*ch*^2^ = 64.013, *df* = 15, *p* < 0.001; top panel in Table 1). The answer “It was terrible, and with distance education, it became even worse” was chosen the least often by the youngest students (5.5% 9–12 years old, 12.0% 13–15 years old, 13.1% 16–17 years old, 12.9% 18–20 years old). The response proving a stable malaise (“It was terrible, and with distance education, it stayed that way”) was chosen more often by adolescents than the youngest and the oldest students (5.5% 9–12 years old, 11.5% 13–15 years old, 10.1% 16–17 years old, 8.4% 18–20 years old). Improvement of well-being from bad to good (“It was terrible, and with distance education, it became good”) was declared by older rather than younger people (5.5% 9–12 years old, 5.9% 13–15 years old, 13.6% 16–17 years old, 12.2% 18–20 years old). Interestingly, the change from good to bad (“It was good, and with distance education, it became terrible”) was declared more often by younger than older people (40.4% 9–12 years old, 38.1% 13–15 years old, 29.5% 16–17 years old, 31.2% 18–20 years old). However, this trend also applied to stable well-being (“It was good, and with distance education, it stayed that way”; 32.4% 9–12 years old, 24.0% 13–15 years old, 24.2% 16–17 years old, 28.1% 18–20 years old) as well as its improvement (“It was good, and with distance education, it became even better”; 10.9% 9–12 years old, 8.5% 13–15 years old, 9.5% 16–17 years old, 7.2% 18–20 years old).

Students of different ages also assessed how they spent their leisure time differently (*ch*^2^ = 40.826, *df* = 15, *p* < 0.001; bottom panel in Table 1). The youngest ones did not choose the answers “It was terrible, and with distance education, it became even worse” (2.9% 9–12 years old, 7.6% 13–15 years old, 7.2% 16–17 years old, 7.2% 18–20 years old) and “It was terrible, and with distance education, it stayed that way” (5.5% 9–12 years old, 8.8% 13–15 years old, 8.9% 16–17 years old, 6.8% 18–20 years old). The answer that was characteristic of them was: “It was good, and with distance education, it stayed that way” (42.2% 9–12 years old, 33.8% 13–15 years old, 26.1% 16–17 years old, 28.9% 18–20 years old). The older the students, the more often they declared that their free time “was terrible, and it became good with distance education” (9.8% 9–12 years old, 14% 13–15 years old, 16.5% 16–17 years old, 17.9% 18–20 years old).

The factors related to the size of the participants’ place of residence and their school did not differentiate students’ responses regarding the assessment of their physical well-being and the way of spending leisure time (all *p* > 0.638). The location of the place of residence (in the countryside, in small towns, in large cities) of students did not differentiate their physical well-being (*ch*^2^ = 4.670, *df* = 10, *p* = 0.912) or their leisure time (*ch*^2^ = 6.826, *df* = 10, *p* = 0.742). There was also no significant relation between the school’s location and the student’s physical well-being (*ch*^2^ = 5.725, *df* = 10, *p* = 0.838) and their leisure time (*ch*^2^ = 7.904, *df* = 10, *p* = 0.638).

### 3.2. Physical Well-Being and Leisure Time—Interactions between Demographic Characteristics

The researched phenomena (students’ physical well-being and leisure time) were significantly related to demographic characteristics: students’ gender and their age. The simple main effects of both factors were confirmed. We ran a regression analysis to check the relationships between each studied phenomenon and the demographic characteristics (gender, age, place of residence, and school location), creating models covering four predictors. The research phenomena were measured with a nominal scale. In each label, the participants compared their experiences, and it is impossible to put them into a specific order. Thus, for a better understanding of how changes in predictors (in demographic characteristics) are associated with the relative odds of choosing the specific category of assessment of physical well-being and the way of spending leisure time, multinomial logistic regression was used (Table 2).

The analysis of the model of relations between the students’ physical well-being and their demographic characteristics as a whole fit significantly better than an empty model (*log-likelihood* (*LL*) = 807.961; *likelihood ratio* (*LR*) *ch*^2^ = 89.083, *df* = 40, *p* < 0.001, *pseudo R*^2^ = 0.054). The analysis of the model showed that the students’ assessment of physical well-being can be explained to the greatest extent by age (*LL* = 871.105; *LR ch*^2^ = 63.145, *df* = 15, *p* < 0.001) and then by gender (*LL* = 827.629; *LR ch*^2^ = 19.669, *df* = 5, *p* < 0.001). Two other elements are statistically insignificant in the model: place of residence (*LL* = 814.886; *LR ch2* = 6.925, *df* = 10, *p* = 0.732) and school location (*LL* = 817.862; *LR ch*^2^ = 9.901, *df* = 10, *p* = 0.449). In relation to gender, the relative log odds of being in the baseline category of physical well-being (“It was terrible, and with distance education, it became even worse”) versus being in one of the other three categories (“It was terrible, and with distance education, it became good”; “It was good, and with distance education, it stayed that way”, or “It was good, and with distance education, it became even better”) increased from 0.500 to 0.607 when differentiating boys from girls. The ratio of choosing one of the outcome categories of physical well-being over the probability of choosing the baseline category was higher among boys than among girls. In relation to age, the relative log odds of being in the baseline category of physical well-being (“It was terrible, and with distance education, it became even worse”) versus being in one of the other three categories (“It was good, and with distance education, it became terrible”; “It was good, and with distance education, it stayed that way”, or “It was good, and with distance education, it became even better”) increased when differentiating the youngest group of students from each of the older groups. The ratio of choosing one of the outcome categories of physical well-being over the probability of choosing the baseline category was higher among the youngest students (9–12 years old) than among the older groups of students.

The multinomial logistic regression analysis model was also used for analyzing the associations between students’ assessments of the way of spending leisure time and their demographic characteristics (gender, age, place of residence, and school location). As a whole, this model fits significantly better than an empty model (*LL* = 838.287; *LR ch*^2^ = 77.328, *df* = 40, *p* < 0.001, *pseudo R*^2^ = 0.047). The model confirmed that the students’ assessment of leisure time is mostly explained by age (*LL* = 875.914; *LR ch*^2^ = 37.628, *df* = 15, *p* < 0.001) and, further, by gender (*LL* = 859.122; *LR ch*^2^ = 20.835, *df* = 5, *p* < 0.001), whereas two other factors: place of residence (*LL* = 848.926; *LR ch*^2^ = 10.640, *df* = 10, *p* = 0.386) and school location (*LL* = 845,125; *LR ch*^2^ = 6.839, *df* = 10, *p* = 0.741) are insignificant. In relation to gender, the relative log odds of choosing the baseline category of leisure time (“It was terrible, and with distance education, it became even worse”) versus choosing the category “It was good, and with distance education, it became even better” increased by 0.566 when differentiating girls from boys (the ratio of choosing baseline category was higher among girls). In relation to age, the relative log odds of being in the baseline category of assessment of leisure time (“It was terrible, and with distance education, it became even worse”) versus being in one of the other three categories (“It was good, and with distance education, it became terrible”; “It was good, and with distance education, it stayed that way”, or “It was good, and with distance education, it became even better”) increased when differentiating the youngest group of students from the older groups. The ratio of choosing the outcome categories over the probability of choosing the baseline category was higher among the youngest students (9–12 years old) in comparison to older groups of students (specifically with 13–15-year-olds).

The multinomial logistic regression model confirmed that age and gender explain the differences in the students’ assessment of researched phenomena, while place of residence and school location are not significant in this matter. The construction of dependable variables (measured on the nominal scale) excludes the direct statistic indicators of the cross-interaction effects of gender and age in relation to physical well-being and way of spending leisure time (e.g., it is not possible to use two-way *ANOVA*). Thus, we ran subgroups analysis (by gender and by age) to check the potential interaction effects (see Table 3).

The analysis conducted in subgroups by gender confirmed the main results of the relation between age and physical well-being. Age differentiated physical well-being among the subgroup of male students (*ch*^2^ = 26.834, *df* = 15, *p* < 0.05) and among the subgroup of female students (*ch*^2^ = 38.167, *df* = 15, *p* < 0.001). Age also differentiated the leisure time of male students (*ch*^2^ = 25.456, *df* = 15, *p* < 0.05) and female students (*ch*^2^ = 32.836, *df* = 15, *p* < 0.05). The differences between the four age groups of males and females corresponded with the already presented main effects. For instance, youngest girls, in comparison to older ones, rarely indicated that their physical well-being was terrible before the pandemic or changed the status of their well-being to terrible, e.g., the answer “it was terrible, and with distance education, it became even worse” (6.5% 9–12 years old, 11.1% 13–15 years old, 13.9% 16–17 years old, 14.7% 18–20 years old). Younger boys, relatively more often than older ones, assessed their way of spending leisure time as stable and good, e.g., the answer “it was good, and with distance education, it stayed that way” (42.2% 9–12 years old, 37.6% 13–15 years old, 26.8% 16–17 years old, 33.3% 18–20 years old).

The analysis conducted in subgroups by age revealed a more complex relationship between gender, physical well-being, and leisure time. The differences between male and female students concerning both factors were consistent with the already described main effects. However, just some of them were significant. Firstly, the gender differences in physical well-being proved significant only for the subgroup of 16–17-year-old students (*ch*^2^ = 17.041, *df* = 5, *p* < 0.01). Female students from this age group described their physical well-being as terrible more often than males, e.g., “It was terrible, and with distance education, it stayed that way” (6.9% of boys and 13.9% of girls); boys more often indicated good well-being, e.g., “It was good, and with distance education, it became even better” (11.5% of boys and 7.5% of girls). Secondly, the gender differences in physical well-being were insignificant in all other age subgroups: 9–12 years old (*ch*^2^ = 2.575, *df* = 5, *p* = 0.765); 13–15 years old (*ch*^2^ = 3.731, *df* = 5, *p* = 0.859), and 18–20 years old (*ch*^2^ = 6.707, *df* = 5, *p* = 0.243). Thirdly, the gender differences of ways of spending leisure time proved to be significant for two age subgroups: 13–15 years old (*ch*^2^ = 15.159, *df* = 5, *p* < 0.01) and 16–17 years old (*ch*^2^ = 13.450, *df* = 5, *p* < 0.05). Boys more often than girls indicated answers showing an increase in the ways of spending leisure time, e.g., “It was good, and with distance education, it became even better” (among 13–15-year-olds: 17.1% of boys and 8.7% of girls; among 16–17-year-olds: 22.1% of boys and 14.6% of girls). In contrast, girls more often than boys indicated a decrease in the ways of spending leisure time; e.g., “It was good, and with distance education, it became terrible” (among 13–15-year-olds: 18.1% of boys and 28.6% of girls; among 16–17-year-olds: 19.3% of boys and 29.5% of girls). Finally, gender differences in leisure time were insignificant between the youngest students (9–12 years old; *ch*^2^ = 5.127, *df* = 5, *p* = 0.401) and the oldest ones (18–20 years old; *ch*^2^ = 6.659, *df* = 5, *p* = 0.247).

## 4. Discussion

Previous studies have indicated differentiation depending on socioeconomic features and an overwhelming cultural, social, and economic stratification of the pandemic’s effects [1,4,13]. In light of socio-historical and developmental concepts and due to the prolonged period of the COVID-19 crisis, long-term consequences for children and adolescents can be expected [13]. Children and adolescents from marginalized environments and less developed countries, who are at risk of exclusion, may face negative consequences [13]. Therefore, it is crucial to research specific aspects of outbreak effects on students from specific groups or countries. The results of our study, the first of such studies done in Poland, demonstrate a significant decrease in the physical well-being of Polish students during the COVID-19 outbreak. While 70% of respondents indicated that their physical well-being before the pandemic was good, half of them experienced a deteriorated state during the time of distance education. Therefore, the outcomes we obtained correspond to previous studies from other countries [10,13,23,24], proving that the pandemic hurts the well-being of children and adolescents. Undoubtedly, it is associated with an observable decrease in physical activity and a marked increase in emotional disorders and problems, as well as a level of stress that can cause psychosomatic symptoms [13,24].

However, a deterioration in well-being did not apply to all respondents. Although almost half of the schoolchildren experienced a deterioration in their physical well-being during the pandemic, more than a third did not see any changes and one in five felt even better than before. The results of an earlier Polish study [25] also indicated that the epidemic situation caused a feeling of discomfort in a large group of respondents. However, almost the same number of people did not feel these problems. Another Polish study [26] showed that although most teenage students (about 40%) declared that they felt much worse or slightly worse than before the pandemic, every sixth student indicated that they felt a little better or even much better. Such positive effects of the pandemic were also observed in studies from other countries [8,10,13,22] and included feeling relieved from social pressure and bullying, spending more time with family and loved ones, taking up new hobbies and creative activities, and spending more time on favorite pleasant activities, such as watching TV programs, listening to music, idling, using social media, and reading. Nevertheless, both our study and previous studies indicate that the group experiencing positive changes in physical well-being is in the minority. Perhaps it includes students who have developed coping strategies and ways to meet basic needs, as well as new forms of activity to keep fit [2].

Interestingly, most of our study participants (6 in 10) assessed that their leisure time activities were suitable during the pandemic (but more before; 7 in 10). Most respondents (6 in 10) also recognized that their quality of free time had changed (the same number of participants perceived a change for the better and for the worse). Thus, our project reveals a relatively high level of change in how the surveyed students perceive their way of spending free time. This observation may result from objectively occurring changes in the way children and adolescents spend their free time during the pandemic, which was confirmed by previous studies [2,3].

In our study, boys rather than girls declared better well-being. Additionally, the issue of free time was assessed as better by boys. These results may support other diagnoses showing that in the course of the pandemic, distance education may promote already privileged groups [27]. Research conducted in Poland by Bieganowska-Skora and Pankowska [25] indicated that girls experienced the general feeling of discomfort more than boys. At the same time, it is worth remembering that there is empirical evidence that men and boys, due to the social norms of traditional masculinity, inhibit emotional expression, which affects, for example, the perception of one’s well-being [28]. As a result, their claims of better well-being can be overstated. In addition, studies of Italian children and adolescents [2] indicate a clear gender difference in changes in leisure activities during the COVID-19 pandemic. Some online activities that may be considered risky (e.g., playing online computer games and exposure to pornographic content) during the pandemic were more often than before undertaken by young (under 25) men (54%—games; 29%—pornography) than women (19% and 7%, respectively). Meanwhile, more young women (51% vs. 43% of men) indicated that during the pandemic, they used online webinars and tutorials more often than before, and similar differences were found in communication and use of social media [2]. These changes can be considered more pro-development than those occurring in the way boys spend time. Hence, the differences in assessing the quality of free time can be compared with the different levels of self-esteem or self-criticism of men and women.

Our outcomes also show that younger students prevailed in the groups declaring stable or increased well-being. An improvement in well-being from bad to good (but also a deterioration from bad to worse) was declared by older students rather than younger students. Stable malaise—both before and during the pandemic—describes most adolescents. Improvement in the ways to spend free time was declared by older students versus younger ones; the latter were more willing to point out that their free time was good and remained that way.

The analysis conducted in subgroups by gender confirmed the main results of the relation between age, physical well-being, and leisure time. The subgroups analysis—run to check the potential interaction effects by age—revealed a more complex relationship between gender, physical well-being, and leisure time. It turns out that gender differences in physical well-being proved significant only for the subgroup of 16–17-year-olds; gender differences in the way of spending leisure time proved significant in the groups of 13–15-year-olds and 16–17-year-olds. Thus, younger age made the gender effect insignificant, and the experiences of girls and boys in adolescence differed the most.

Other studies indicate that gender differences in well-being are observed in adolescents but not at a younger age, suggesting the existence of a gender gap that begins at the age of 12 or 13 [29,30]. This observation also applies to the time of the COVID-19 outbreak. Polish studies on adolescents have obtained data on the deterioration of the well-being of adolescents; this is more frequent in girls than in boys [31].

Additionally, the differences in ways of spending free time between girls and boys in adolescence become evident. Younger students spend their free time on electronic media and sports more often and adolescents on socializing and relaxing activities [32]. Older girls become less sports-active than boys in leisure-time activities [33,34]; their way of spending free time can be described as more social. Perhaps it can be a way of explaining why teenage girls are a group that perceives their leisure time in a pandemic as worse than before. The social dimension of life was particularly disrupted during the COVID-19 outbreak, affecting more adolescent girls than other student age groups or boys.

In Poland, rural schools and communities, on the one hand, coped better during the pandemic than the city schools (fewer students and a more extensive network of informal contacts between teachers and the local community). On the other hand, they coped worse (deficits related to a lack of computer equipment, poor access to high-speed internet, and a lack of digital competencies) [35]. Thus, we decided to check the relationships between place of residence and school location and well-being and ways of spending free time. In our study, these environmental variables did not differentiate student experiences regarding the assessment of their physical well-being and the ways of spending leisure time.

## 5. Conclusions

In light of our study results, we recommend parents and teachers to: (1) strengthen students in the effective use of their own skills and resources for spending free time (e.g., higher than in adults’ media competences, which allow for the maintenance of relationships and the development of new patterns of spending free time) [2,3]; (2) develop learning techniques to identify the signals of emotional distress and the effects of contact with risk factors in both the mental and physical spheres [2,5]; (3) increase pressure to develop individual leisure and physical activity strategies and to support the transformation of entertainment and sports institutions so that they can operate during pandemic times [3,5]; (4) counteract technostress and information overload by strengthening the selective and critical reception/filtering of media information, strengthening self-regulation processes, and developing strategies of using media, e.g., blocking undesirable content and developing applications that allow for self-control of the time devoted to various forms of media activity [8,10].

Our study confirms the existence of demographic differences in the students’ perception of how pandemics affect physical well-being and the quality of leisure time. We also found that some groups should receive exceptional support to overcome significant differences. A program for restoring well-being after the pandemic and supporting pro-development ways of spending free time among adolescents is needed, with particular consideration for gender differences. Girls, especially in adolescence, need tailored assistance.

## Figures and Tables

**Table 1:** Dependent results of the study.

**Physical Well-Being**	**Gender** ***N* = 1749**		**Age** ***N* = 1783**			
	**Boys** ***N* = 903**	**Girls** ***N* = 846**	**9–12** ***N* = 275**	**13–15** ***N* = 591**	**16–17** ***N* = 654**	**18–20** ***N* = 263**
It was terrible, and with distance education, it became even worse	9.1%	11.3%	5.5%	12.0%	13.1%	12.9%
It was terrible, and with distance education, it stayed that way	7.3%	11.0%	5.5%	11.5%	10.1%	8.4%
It was terrible, and with distance education, it became good	10.6%	9.0%	5.5%	5.9%	13.6%	12.2%
It was good, and with distance education, it became terrible	33.3%	36.5%	40.4%	38.1%	29.5%	31.2%
It was good, and with distance education, it stayed that way	29.7%	24.6%	32.4%	24.0%	24.2%	28.1%
It was good, and with distance education, it became even better	10.0%	7.6%	10.9%	8.5%	9.5%	7.2%
**Leisure Time**	**Gender** ***N* = 1749**		**Age** ***N* = 1783**			
	**Boys** ***N* = 903**	**Girls** ***N* = 846**	**9–12** ***N* = 275**	**13–15** ***N* = 591**	**16–17** ***N* = 654**	**18–20** ***N* = 263**
It was terrible, and with distance education, it became even worse	6.1%	7.0%	2.9%	7.6%	7.2%	7.2%
It was terrible, and with distance education, it stayed that way	8.1%	7.2%	5.5%	8.8%	8.9%	6.8%
It was terrible, and with distance education, it became good	13.7%	15.2%	9.8%	14.0%	16.5%	17.9%
It was good, and with distance education, it became terrible	20.0%	26.6%	22.5%	22.3%	23.4%	24.3%
It was good, and with distance education, it stayed that way	33.6%	30.9%	42.2%	33.8%	26.1%	28.9%
It was good, and with distance education, it became even better	18.5%	13.1%	17.1%	13.4%	17.9%	14.8%

**Table 2:** Results of multinomial logistic regression.

**Physical Well-Being**															
	It was terrible, and with distance education, it stayed that way			It was terrible, and with distance education, it became good			It was good, and with distance education, it became terrible			It was good, and with distance education, it stayed that way			It was good, and with distance education, it became even better		
	*OR*	*SE*	*p*	*OR*	*SE*	*p*	*OR*	*SE*	*p*	*OR*	*SE*	*p*	*OR*	*SE*	*p*
**Cons.**	0.304	0.443	0.493	−0.045	0.448	0.920	2.018	0.344	<0.001	1.824	0.350	<0.000	0.515	0.412	0.211
**Gender (RC—Girls)**	−0.089	0.226	0.693	0.500	0.223	0.025	0.268	0.177	0.130	0.525	0.183	0.004	0.607	0.229	0.008
**Age (RC—9–12)**															
13–15	−0.040	0.424	0.925	−0.553	0.437	0.206	−0.691	0.323	0.033	−1.008	0.331	0.002	−1.039	0.387	0.007
16–17	−0.305	0.432	0.480	0.190	0.429	0.657	−1.187	0.332	<0.001	−1.184	0.338	<0.001	−0.997	0.391	0.011
18–20	−0.369	0.493	0.455	0.222	0.477	0.642	−0.996	0.377	0.008	−0.923	0.382	0.016	−1.113	0.462	0.016
**Place of Residence (RC—Large City)**															
Countryside	−0.160	0.423	0.705	−0.572	0.413	0.167	0.134	0.332	0.686	−0.144	0.343	0.674	−0.057	0.427	0.894
Small Town	−0.208	0.443	0.638	−0.240	0.430	0.577	0.101	0.349	0.772	−0.137	0.361	0.705	0.100	0.446	0.823
**School Location (RC—Large City)**															
Countryside	−0.623	0.507	0.219	0.367	0.483	0.447	−0.560	0.377	0.137	−0.398	0.393	0.312	−0.217	0.487	0.656
Small Town	0.101	0.376	0.789	0.071	0.370	0.849	−0.165	0.293	0.572	−0.031	0.304	0.919	−0.063	0.377	0.868
**Leisure Time**															
	It was terrible, and with distance education, it stayed that way			It was terrible, and with distance education, it became good			It was good, and with distance education, it became terrible			It was good, and with distance education, it stayed that way			It was good, and with distance education, it became even better		
	*OR*	*SE*	*p*	*OR*	*SE*	*p*	*OR*	*SE*	*p*	*OR*	*SE*	*p*	*OR*	*SE*	*p*
**Cons.**	−0.135	0.521	0.796	0.875	0.459	0.057	1.611	0.427	<0.001	2.191	0.413	<0.001	1.035	0.444	0.020
**Gender (RC—Girls)**	0.338	0.266	0.204	0.070	0.235	0.765	−0.097	0.221	0.659	0.296	0.215	0.168	0.566	0.233	0.015
**Age (RC—9–12)**															
13–15	−0.410	0.501	0.413	−0.575	0.453	0.204	−0.806	0.420	0.045	−1.090	0.407	0.007	−1.131	0.435	0.009
16–17	−0.288	0.511	0.573	−0.333	0.460	0.469	−0.592	0.430	0.168	−1.274	0.418	0.002	−0.691	0.441	0.117
18–20	−0.438	0.577	0.448	−0.235	0.510	0.645	−0.593	0.479	0.215	−1.188	0.466	0.011	−1.000	0.499	0.045
**Place of Residence (RC—Large City)**															
Countryside	0.825	0.488	0.091	0.617	0.433	0.154	0.392	0.416	0.346	0.568	0.402	0.158	0.516	0.435	0.236
Small Town	1.355	0.515	0.090	0.839	0.463	0.070	0.691	0.443	0.119	0.688	0.432	0.111	0.537	0.466	0.249
**School Location (RC—Large City)**															
Countryside	−0.269	0.592	0.650	−0.418	0.542	0.441	0.236	0.508	0.642	−0.113	0.494	0.819	−0.019	0.535	0.971
Small Town	−0.561	0.442	0.205	−0.385	0.404	0.339	−0.131	0.390	0.737	−0.249	0.380	0.512	−0.020	0.407	0.961

The “It was terrible, and with distance education, it became even worse” category was used as the baseline category for comparisons; *OR*—log odds ratio; *SE*—standard error of the log odds ratio; *RC*—reference category; statistically significant effects were highlighted in black. *N* = 1660.

**Table 3:** Dependent results of the study in subgroups by age and by gender.

**Physical Well-Being**	**9–12** ***N* = 255**		**13–15** ***N* = 539**		**16–17** ***N* = 616**		**18–20** ***N* = 250**	
	**Boys** ***N* = 116**	**Girls** ***N* = 139**	**Boys** ***N* = 287**	**Girls** ***N* = 252**	**Boys** ***N* = 321**	**Girls** ***N* = 295**	**Boys** ***N* = 141**	**Girls** ***N* = 109**
It was terrible, and with distance education, it became even worse	4.3%	6.5%	10.1%	11.1%	9.7%	13.9%	7.1%	14.7%
It was terrible, and with distance education, it stayed that way	5.2%	5.8%	9.4%	11.5%	6.9%	13.9%	7.1%	10.1%
It was terrible, and with distance education, it became good	6.0%	5.0%	7.7%	4.8%	14.3%	12.5%	12.1%	12.8%
It was good, and with distance education, it became terrible	39.7%	38.8%	38.0%	41.7%	28.7%	31.2%	31.9%	32.1%
It was good, and with distance education, it stayed that way	31.0%	35.3%	26.8%	23.0%	29.0%	21.0%	34.0%	22.9%
It was good, and with distance education, it became even better	13.8%	8.6%	8.0%	7.9%	11.5%	7.5%	7.8%	7.3%
**Leisure Time**	**9–12** ***N* = 255**		**13–15** ***N* = 539**		**16–17** ***N* = 616**		**18–20** ***N* = 250**	
	**Boys** ***N* = 116**	**Girls** ***N* = 139**	**Boys** ***N* = 287**	**Girls** ***N* = 252**	**Boys** ***N* = 321**	**Girls** ***N* = 295**	**Boys** ***N* = 141**	**Girls** ***N* = 109**
It was terrible, and with distance education, it became even worse	5.2%	1.4%	7.0%	7.9%	6.2%	7.1%	3.5%	11.0%
It was terrible, and with distance education, it stayed that way	6.0%	4.3%	7.7%	8.7%	10.0%	6.8%	7.1%	7.3%
It was terrible, and with distance education, it became good	6.9%	12.2%	12.5%	13.9%	15.6%	16.3%	17.0%	18.3%
It was good, and with distance education, it became terrible	22.4%	22.3%	18.1%	28.6%	19.3%	29.5%	24.8%	23.9%
It was good, and with distance education, it stayed that way	42.2%	43.9%	37.6%	32.1%	26.8%	25.8%	33.3%	24.8%
It was good, and with distance education, it became even better	17.2%	15.8%	17.1%	8.7%	22.1%	14.6%	14.2%	14.7%

## Data Availability

The data presented in this study are available on request from the author who carried out the analyses (M.M.). The data are not publicly available due to information that could compromise the anonymity of the research participants.
